# Impact of hypoxia stress on the physiological responses of sea cucumber *Apostichopus japonicus*: respiration, digestion, immunity and oxidative damage

**DOI:** 10.7717/peerj.4651

**Published:** 2018-04-27

**Authors:** Da Huo, Lina Sun, Xiaoshang Ru, Libin Zhang, Chenggang Lin, Shilin Liu, Xiaoke Xin, Hongsheng Yang

**Affiliations:** 1 CAS Key Laboratory of Marine Ecology and Environmental Sciences, Institute of Oceanology, Chinese Academy of Sciences, Qingdao, China; 2 Laboratory for Marine Ecology and Environmental Science, Qingdao National Laboratory for Marine Science and Technology, Qingdao, China; 3 University of Chinese Academy of Sciences, Beijing, China

**Keywords:** Echinoderm, Anoxia, Metabolism, Aquatic environment, Dissolved oxygen, Physiological behavior

## Abstract

Hypoxia is one of the most frequently occurring stressors confronted by industrial cultures of sea cucumber and can cause large economic losses and resource degradation. However, its responsive mechanisms are still lacking. In this paper, the physiological responses of *Apostichopus japonicus* to oxygen deficiency was illustrated, including induced oxidative response and immune defense and changed digestive enzymes activities. Significantly increased activities of alpha-amylase (AMS), acid phosphatase (ACP), lactate dehydrogenase, catalase, peroxidase, succinate dehydrogenase and higher content of malondialdehyde, and decreased activities of lipase and trypsin (TRY) were observed after hypoxia exposure (dissolved oxygen [DO] 2 mg/L). Expressions of key genes showed that *AMS, peptidase, ACP, alkaline phosphatase, lysozyme, heat shock protein 70* and *glutathione peroxidase* were increased and *TRY* was decreased under hypoxia. With the decline of the DO level, the decreased tendency of oxygen consumption rates was different in varied weight groups. Moreover, respiratory trees were observed degraded under long-term hypoxia stress, thus leading a negative effect of respiration. These results could help to develop a better understanding of the responsive mechanism of sea cucumber under hypoxia stress and provide a theoretical basis for the prevention of hypoxia risk.

## Introduction

In aquatic systems, hypoxia stress is often defined as dissolved oxygen (DO) levels below 2 mg/L (Wu, 2002). DO deficiency can affect aquatic animals in adverse ways, including a reduction in growth and reproduction, and can even be lethal in sensitive organisms (Huang et al., 2012). The sea cucumber *Apostichopus japonicus* is an important commercial species distributed mainly along the coast of Asian countries (Liao, 1997). Aquaculture of sea cucumbers has grown rapidly in recent years, with around 90,000 tons harvested in China every year (Wang et al., 2005, 2008). However, oxygen deficiency in summer is a chief cause of extensive mortality in recent years, resulting in severe economic and resource losses. These problems strongly limit and challenge the development of the sea cucumber industry. It is becoming a serious issue noticed by fishermen, scientists, and the society.

Over the last few years, more studies have investigated the effect of low levels of DO on marine species such as sea bass (Terova et al., 2008), Japanese medaka (Ju et al., 2007), Pacific oyster (David et al., 2005; Sussarellu et al., 2013), grass shrimp (Li & Brouwer, 2009), Indian catfish (Mohindra et al., 2015), and euryoxic fish (Gracey, Troll & Somero, 2001). In addition, hypoxia resistance in echinoderms is starting to be uncovered. For example, reduced arm growth, decreased arm regeneration rate and disturbance of spawning were demonstrated in the brittle star *Amphiura filiformis* under a hypoxic environment (Nilsson & Sköld, 1996; Nilsson, 1999). After hypoxia exposure, total coelomocyte count and expression of heat shock protein 70 (HSP70) and Runt gene were highly elevated in sea stars *Asterias rubens* (Holm, Hernroth & Thorndyke, 2008; Oweson et al., 2010). Moreover, in sea urchin, changed acid–base status, suppressed gonad growth and decreased total food consumption were observed in the hypoxic group (Spicer, 1995; Siikavuopio et al., 2007). Metabolic compensation in hairy sea cucumber *Sclerodactyla briareus* under the oxygen deficiency condition has also been studied (Ellington & Hammen, 1977). The molecular responses of sea cucumber *A. japonicus* under hypoxia exposure have been partially reveled by constructing gene and miRNA expression profile (Huo et al., 2017; Zhang et al., 2017). However, to our knowledge, the physiological and behavioral responses following hypoxia stress in the holothurian species have not been fully studied until now. Moreover, compared with other marine species, which can survive in the absence of oxygen for periods ranging from several days to a few weeks, the tolerance range of echinoderms is relatively narrow (Riedel, Zuschin & Stachowitsch, 2012). In which case, massive mortality could easily occur to sea cucumber, when hypoxia lasts for several days. Hence, the sea cucumber is an important model for studying stress responses under hypoxia and its mechanisms to cope with hypoxia urgently need to be uncovered.

To cope with hypoxia stress, a series of molecular, physiological and morphological changes are expected to occur in *A. japonicus* for survival. However, the specific responses to hypoxia stress are not well understood. Therefore, in this study, we summarized the physiological behavioral responses of sea cucumbers exposed to DO deficiency, including physical status, organoleptic characteristics and movement. Moreover, we analyzed several indexes focusing on different activities of specific enzymes, expression of related genes, oxygen consumption rates (OCRs), and histomorphology of the respiratory tree to reveal the responsive mechanisms of *A. japonicus* under hypoxia stress, including changes in respiration, digestion, immunity, and oxidative response. The results investigated in this study will provide new insights into understanding the stress responses of *A. japonicus* when exposed to low DO environments and provide a theoretical basis for further study.

## Materials and Methods

### Animals

*Apostichopus japonicus* were supplied by East Ocean Science and Technology Co., Ltd (Shandong, China). The sea cucumbers were transported to laboratory and acclimated in tanks containing aerated sand-filtered seawater (30‰ salinity, 16 ± 0.5 °C) for one week before use and were fed once a day at 11:00 h. Remaining feed was removed daily during the acclimation and experimental periods. Active sea cucumbers were randomly selected from acclimation tanks for further experiments. For experiments of enzymes activities and key genes expression, three DO levels (2, 4, and 8 mg/L) were set. Sea cucumbers (wet weight: 100–120 g) kept in sea water with sufficient DO level at 8 mg/L (DO8), were regarded as the control group, and sea cucumbers kept in sea water with DO level at 4 mg/L (DO4) and 2 mg/L (DO2) were regarded as treatment groups. All the sea cucumbers from each group were cultivated in separated tanks according to the number of biological replicates and last for three days, respectively. Observation of behavioral responses were carried on during these processes. After that, sea cucumbers were selected and dissected promptly, and anatomical observation was carried on at the same time. The coelomic fluids (five biological replicates per group) and respiratory trees (three biological replicates per group) were sampled to be preserved in liquid nitrogen and stored at −80 °C for enzyme assays and RNA extraction, respectively. For experiments of OCRs, four DO levels (2, 4, 6, and 8 mg/L) and two specifications (high weight: 110.42 ± 13.50 g; low weight: 46.15 ± 5.90 g, *P* < 0.01) were set. Details were described in “Respiration Measure.” For experiments of histological observation, two DO levels (2 and 8 mg/L) were set. Sea cucumbers (wet weight: 100–120 g) were separated into three tanks per group, and maintained for 12 days, respectively. After that, three sea cucumbers in each group were dissected promptly, the respiratory trees were sampled to be preserved in paraformaldehyde solution for 24 h before obtaining tissue slices.

### DO regulation

**Figure 1:**
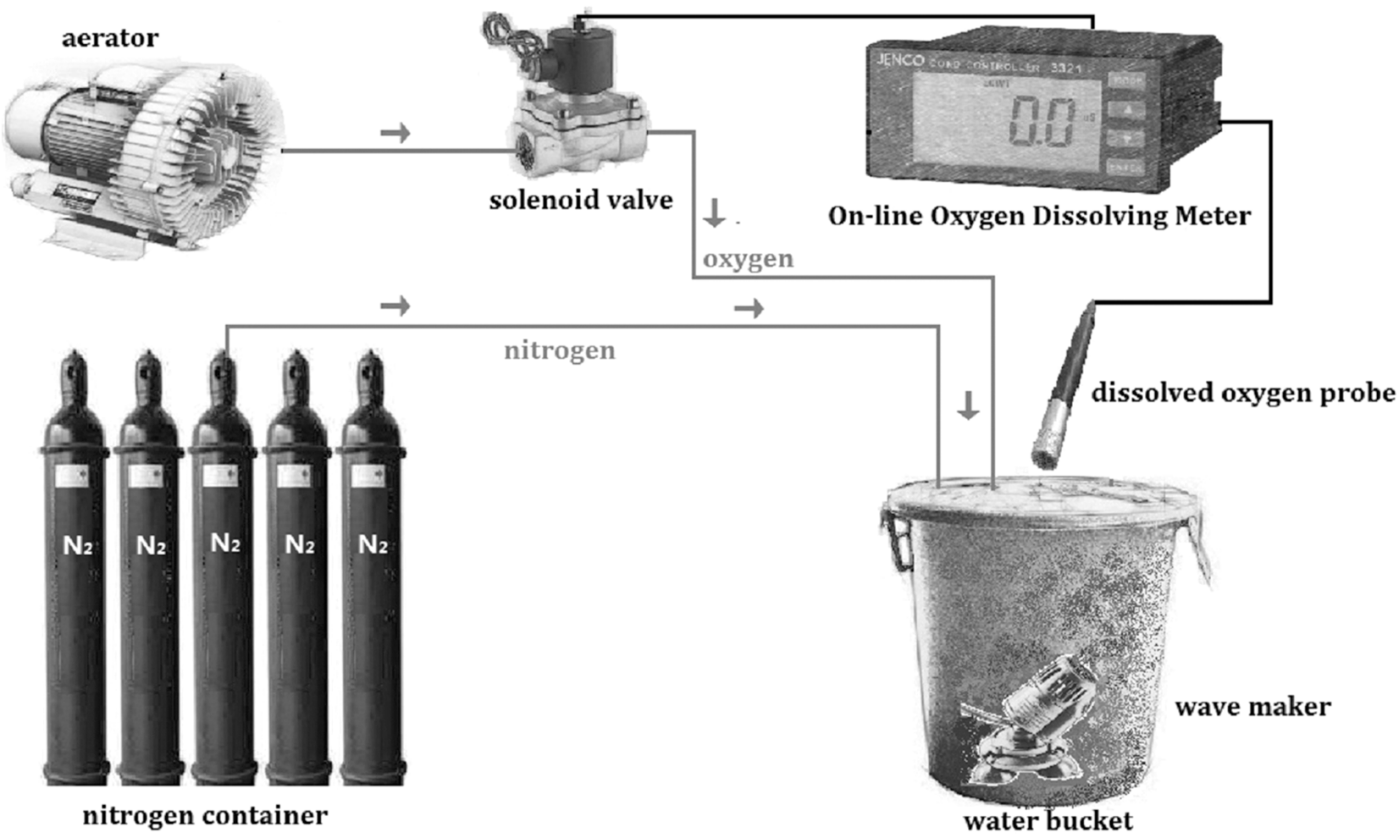
Schematic diagram of the dissolved oxygen control system. The system was constructed by aerator, solenoid valve, on-line oxygen dissolving meter, dissolved oxygen probe, wave maker, nitrogen container and water bucker.

The regulation of DO was by pumping nitrogen into water and then oxygen gradually decreased to the level needed. The traditional method is to use plastic film covering the water bucket after aeration in order to avoid vapor exchange. Occasionally, measurements are needed and then either nitrogen or oxygen is pumped into the experimental aquatic environment artificially to regulate DO level. The DO control system used in this study (Fig. 1) includes aerator, solenoid valve, nitrogen container, wave maker, water bucket, and an online oxygen dissolving meter (JECO6308DT, Wellingborough, UK). The online oxygen dissolving meter was calibrated by the Winkler method (Strickland & Parison, 1968). After setting a specific DO level, the oxygen and nitrogen were pumped into water automatically and stopped when approaching the level set. This can achieve real-time monitoring, improve precision and stability and reduce labor expenses to satisfy the needs of experiments. This system has been successfully used in the experiments of the present study.

### Enzyme activity tests

The activities of enzymes in coelomic fluid of *A. japonicus* were measured in the present study. We focused on the coelomic fluid because it contains bactericidal humoral factors, which are important in host defense against pathogens and other foreign substances, including lectins, agglutinins, and lysins (Gross et al., 1999). Sixteen indexes, involving digestion, oxidation, and immunity, were tested in this study and five biological replicates were set per group. The coelomic fluid of *A. japonicus* was withdrawn immediately with a syringe, frozen in sterile test tubes in liquid nitrogen, and stored for less than one month at–80 °C until analyses. The specific tests used in this study were detected by commercial kits from Nanjing Jianchen Biological Institute (Nanjing, China), which are described in Table 1. Statistical analysis was performed by SPSS19 software (IBM Corp., Armonk, NY, USA) and all the data were indicated as mean ± SD. Significance between different groups of each enzyme was analyzed by one-way analysis of variance (ANOVA) with Tukey’s test, and the threshold of statistical significance was set *P* < 0.05. Statistical graphs were performed using SigmaPlot software for Windows, version 10.0 (Systat, San Jose, CA, USA).

**Table 1:** Selected enzymes and their detection methods.

No.	Gene name	Abbreviation	Detection methods of commercial kit
1	Lipase	LPS	Colorimetry (triglyceride)
2	Alpha-amylase	AMS	Colorimetry (Iodine-Starch)
3	Peptidase	PEP	Colorimetry
4	Trypsin	TRY	Ultraviolet colorimetry
5	Superoxide dismutase	SOD	Method of xanthine oxidase
6	Glutathione peroxidase	GSH-PX	5,5’-dithiobis-2-nitrobenzoic acid colorimetric
7	Catalase	CAT	Ammonium molybdate colorimetry
8	Succinate dehydrogenase	SDH	Colorimetry
9	Lactate dehydrogenase	LDH	2,4-Dinitrophenylhydrazine method
10	Total antioxidant capacity	T-AOC	Phenanthroline colorimetry
11	Acid phosphatase	ACP	*p-*Nitrophenyl sodium phosphate assay
12	Alkaline phosphatase	AKP	Disodium phenyl orthophosphate
13	Lysozyme	LZM	Turbidimetry
14	Malondialdehyde	MDA	Thiobarbituric acid colorimetric assay
15	Peroxidase	POD	Colorimetry
16	Phenoloxidase	PPO	Spectrophotometry

### Real-time polymerase chain reaction validation

Real-time polymerase chain reaction (PCR) was performed in order to illustrate the expression of some important genes related to digestive function, oxidative response, and immune defense. Total RNA was extracted from the respiratory tree, the major tissue responsible for respiration and metabolism under unsuitable conditions, by using MiniBEST Universal RNA Extraction Kit (TaKaRa, Kusatsu, Japan) following the manufacturer’s instructions and three biological replicates were set per group. The quality and concentration of RNA were measured with NanoDrop 1000 (ThermoFisher Scientific, San José, CA, USA). First-strand cDNA was synthesized as the quantitative polymerase chain reaction (qPCR) template using reverse transcriptase (TaKaRa, Kusatsu, Japan). The mRNA expression levels were examined using a SYBR Green® real-time PCR assay (SYBR PrimeScript™ RT-PCR Kit II; TaKaRa, Kusatsu, Japan) with an Eppendorf Mastercycler® ep realplex (Eppendorf, Hamburg, Germany). The gene β-actin was used as a reference gene for internal standardization.

The expression of 11 genes were measured, including *ACP, AKP, AMS, SOD, LDH, PEP, GSH-PX, LZM, TRY, SDH* and *HSP70.* Primers (Table S1) were designed for optimal performance with primer 3 according to the sequence information in the transcriptome database. The total volume of the amplification was 20 μL, which contained 8 μL of RNase-free water, 10 μL of SYBR GreenMasterMix (TaKaRa, Kusatsu, Japan), 1 μL of diluted cDNA, 0.5 μL (each) of forward and reverse primer (10 mM). The thermal cycling was performed according to the following procedure: 95 °C for 5 s, followed by 40 cycles at 95 °C for 10 s, 60 °C for 20 s, and 72 °C for 30 s. The specificity of the amplification products was determined by melting curve analysis. The 2^−ΔΔCT^ method was used to analyze the comparative mRNA expression levels of the selected genes (Schmittgen & Livak, 2008). The statistical analysis was performed with SPSS19 software. All the data were indicated as mean ± SD. Significance analysis between different groups of each gene was analyzed with one-way ANOVA with Tukey’s test, and the threshold of statistical significance was *P* < 0.05.

### Histology observation

After 24 h in paraformaldehyde solution, the respiratory tree of sea cucumbers was preserved in 70% ethanol. Samples were dehydrated in a graded series (70%, 75%, 85%, 95%, and 100%) of ethanol, rinsed with xylene, and then embedded in paraffin. Sections of 5 μm were placed on slides coated with polylysine, then stained with hematoxylin and eosin (Xu et al., 2015). Light microscopy was used to observe tissue sections, and thickness was measured by ImageJ software (Rasband, 1997). Three random points in each tissue section were selected to measure the thickness. The mean of measure results was calculated as the layer thickness of this individual. Three individuals were analyzed in each group.

### Respiration measure

The OCR of the sea cucumbers with two different weight specifications was measured in four DO levels in the present study, and were calculated from the following equation (Makoto & Tsutomu, 1985):

$${\rm{OCR}}\left( {{\rm{mg}}{O_2}{\rm{}}{h^{ - 1}}{g^{ - 1}}} \right) = \left( {{D_t}{V_t} - {D_0}{V_0}} \right)/WT$$

where

*D*_t_: the changes of in oxygen content (mg/L) before and after the test in the test bottles;
*D*_0_: the changes of in oxygen content (mg/L) before and after the test in the blank bottles;
*V*_t_: the volumes of the test bottles (L);
*V*_0_: the volumes of the blank bottles (L);
*W*: the wet weight of the sea cucumber (g); and
*T*: the time duration (h).

In the four DO treatments (DO2, DO4, DO6, and DO8), there were five replicates and two blank controls to correct for the respiration of bacteria in the water. The tested animals were put into a 1 L conical flask individually. Oxygen consumption was determined over 1 h. The oxygen content of water samples was determined using a portable DO meter (YSI ODO200CC-04; Yellow Springs, OH, USA), which was calibrated by the Winkler method (Strickland & Parison, 1968). No animals were eviscerated or died during this procedure.

## Results

### Physiological behavioral responses

In this study, the behavioral and morphological changes of sea cucumbers was observed in normoxic (8 mg/L) and hypoxic conditions (2 mg/L) (Figs. S1 and S2). When DO was around 2 mg/L, the sea cucumbers showed different performance compared with the normoxic condition, including physical status, organoleptic characteristics, and movement. The behavioral responses were summarized in Table 2 according to our observation in the experimental sea cucumbers under hypoxia stress within three days.

**Table 2:** Physiological behavioral responses of sea cucumbers under hypoxia stress.

	Hypoxic condition (2 mg/L)	Normoxic condition (8 mg/L)
***Physical status***		
Body condition	Distorted; fatigued and weak	Active
Body type	Shrunken body; Edematous body with excessive seawater inside the body	Same as the origin
Tentacles	Stretched for a long time and then could not smoothly expand and contract	Stretched regularly and could expand and contract tactile smoothly
Heads	Heads shook frequently	Seldom shook heads
Mouth	Swollen mouths	Normal mouths
Spines	Became white and weak	Straight and hard
Body wall	Ulcerated skin and dissolved	Solid with no ulceration
Longitudinal muscles	Thinner and broken	Normal thickness and complete
Internal organs	Diffused; eviscerated; mid-eviscerated with internal organs hung around the anus.	Complete internal organs
***Organoleptic characteristics***		
Smell	Stenchful smell	No special smell
Color	The body became discolored	Same as original color (green in this study)
***Movement condition***		
Distribution status	Mainly distributed near the surface of the water	Random distributed in the water
Speed of movement	Slower speed	Normal speed
Sticking ability	Weak ability as easy to fall to the bottom when touched	Strong ability

### Respiratory changes

#### Tissue degradation was observed in the respiratory tree

**Figure 2:**
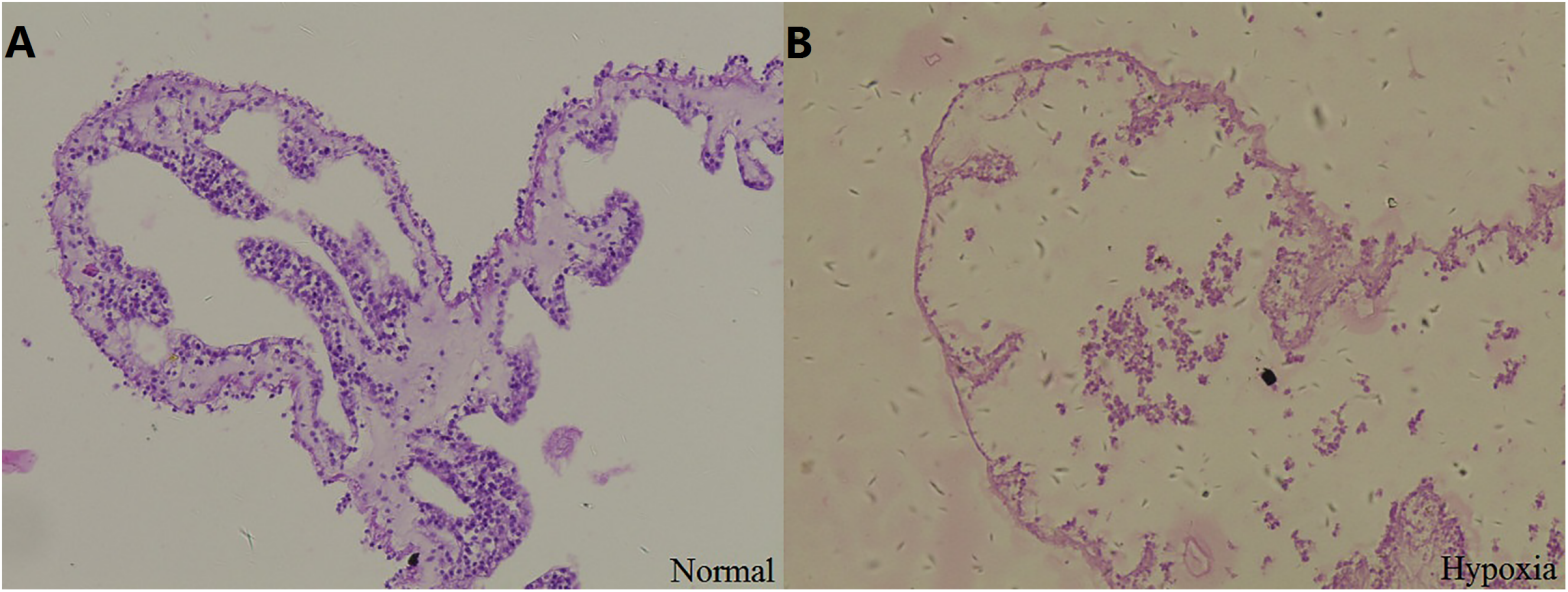
Tissue section of respiratory tree in sea cucumber under long-term hypoxia. (A) respiratory tree of sea cucumber under normal conditions; (B) respiratory tree of sea cucumber under hypoxia.

Stained sections were examined by light microscopy to observe the structural changes in the respiratory tree under hypoxia (Fig. 2). After 12 days, the thickness of the respiratory tree was 17.67 ± 8.12 μm in the control group (8 mg/L) and 4.11 ± 1.97 μm in the hypoxic treatment (2 mg/L). The change in the thickness of respiratory tree was extremely significant (*P* < 0.01).

In addition, changes of internal organs in sea cucumber exposed to hypoxia for three days were observed after dissection (Fig. S3). The color of normal intestines and respiratory trees are primrose yellow, and the respiratory trees should be half-transparent with sufficient water in the normal state. After hypoxia, the respiratory trees and intestines were diffused. The color of fecal matter in intestines appeared black with blurred sections. The moisture condition and color of internal organs were changed under hypoxic stress. Thus, we speculated that hypoxia had a negative effect on the structure and function of organs in sea cucumbers, including respiratory trees and intestines, resulting in a negative effect on the respiratory and digestive function.

#### Decreased OCRs under low dissolved oxygen levels

**Figure 3:**
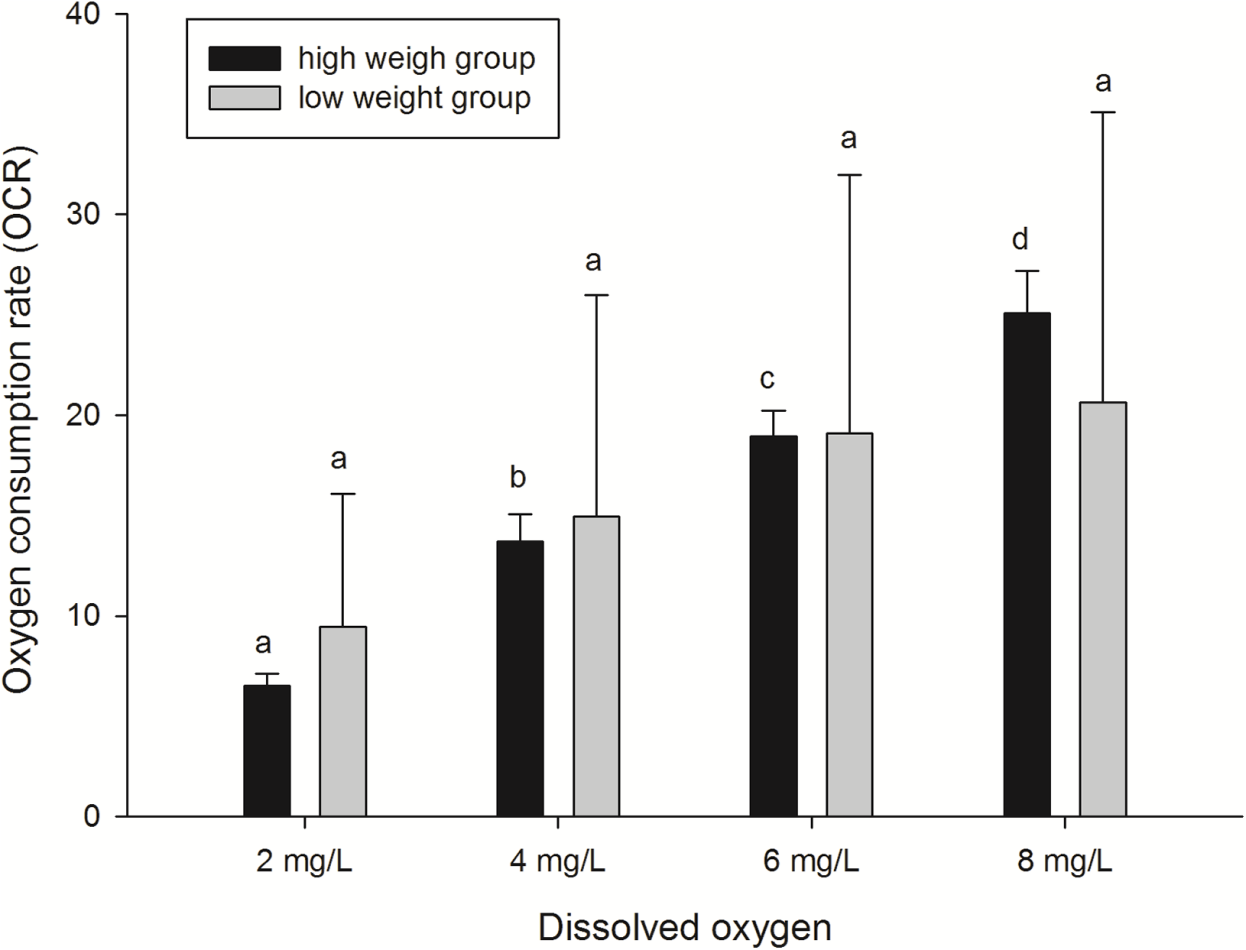
Oxygen consumption rates of *A. japonicus* at different dissolved oxygen levels.

In this study, the effects of different DO levels and body weights on oxygen consumption of sea cucumbers were assessed. OCRs of animals decreased in an oxygen-deficient aquatic environment (DO 2, 4, and 6 mg/L) compared with normoxic conditions (8 mg/L) (Fig. 3). Significant differences (*P* < 0.05) were found among OCRs at different DO levels in the high weight group. While from the aspect of OCRs in the low weight group, the extent of decrease was not significant. As the results show, OCR of the high weight group decreased more rapidly than the low weight group with the decline of DO level. In general, the analysis demonstrated that the effect of DO level on OCR was significant, while the effect of body weight on OCR was not significant. No significant interaction on OCR between body weight and DO level was observed (Table 3). The results indicated that hypoxia suppressed respiration and the negative effect was more obvious in high weight group of sea cucumbers.

**Table 3:** Effect of dissolved oxygen (DO) levels (2, 4, 6, and 8 mg/L) on the oxygen consumption rate (OCR) of the sea cucumber in different body weight (high and low).

Source	Type III sum of squares	df	Mean square	F	P
DO level	1229.489	3	892.473	7.177	0.016
Body weight	0.009	1	0.009	0.000	0.993
DO level × Body weight	75.117	3	54.526	0.438	0.584

### Enzyme activities changes

#### Enzymes associated with digestion

**Figure 4:**
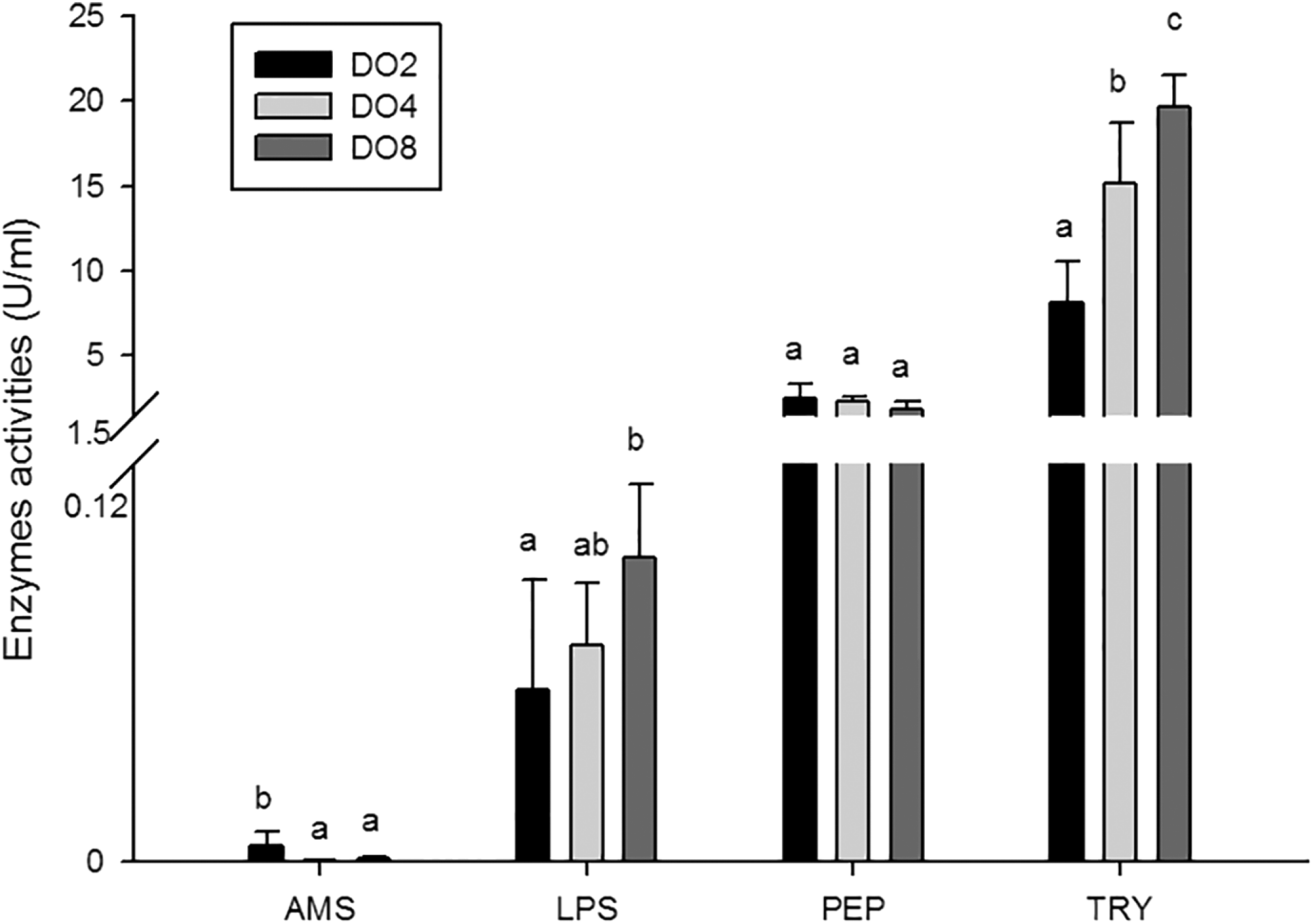
Activities of digestive enzymes in sea cucumber under different dissolved oxygen levels.

In the present study, the activities of four enzymes associated with digestion were selected to be measured (Fig. 4), including lipase (LPS), alpha-amylase (AMS), peptidase (PEP) and trypsin (TRY). The results showed that the enzymatic activities of LPS and TRY were decreased with the decline of DO level and the differences were significant. In addition, the activity of AMS significantly increased under severe hypoxia (DO 2 mg/L). However, activities of PEP were changed without significance in three groups. Thus, the level of DO has an influence on digestive enzymes activities, indicating that hypoxia may have an effect on the digestive function in sea cucumbers.

#### Enzymes associated with immune defense

**Figure 5:**
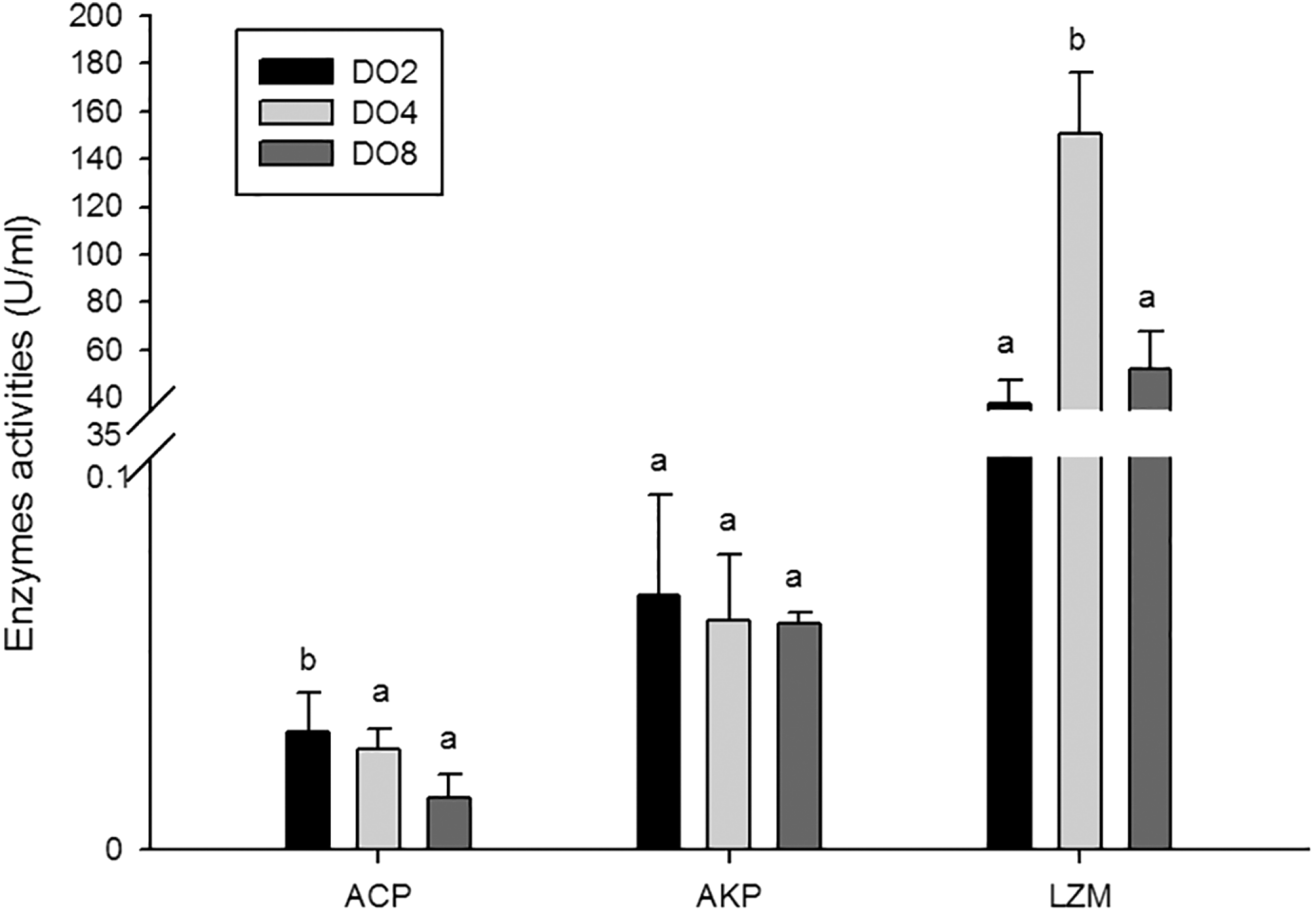
Activities of immune enzymes in sea cucumber under different DO levels.

The activities of three enzymes associated with immune defense were measured in this study (Fig. 5), including acid phosphatase (ACP), alkaline phosphatase (AKP), and lysozyme (LZM). The activity of ACP was significantly higher in severe hypoxia. LZM showed a significantly high level in the DO4 group (4 mg/L), indicating that mild hypoxia stress could lead to a high LZM level in sea cucumber. AKP showed statistically insignificant activities among three groups. Hence, enzyme activity in the coelomic fluid will reflect changes in DO level and oxygen deficiency potentially influence immune activity.

#### Enzymes associated with oxidative stress

**Figure 6:**
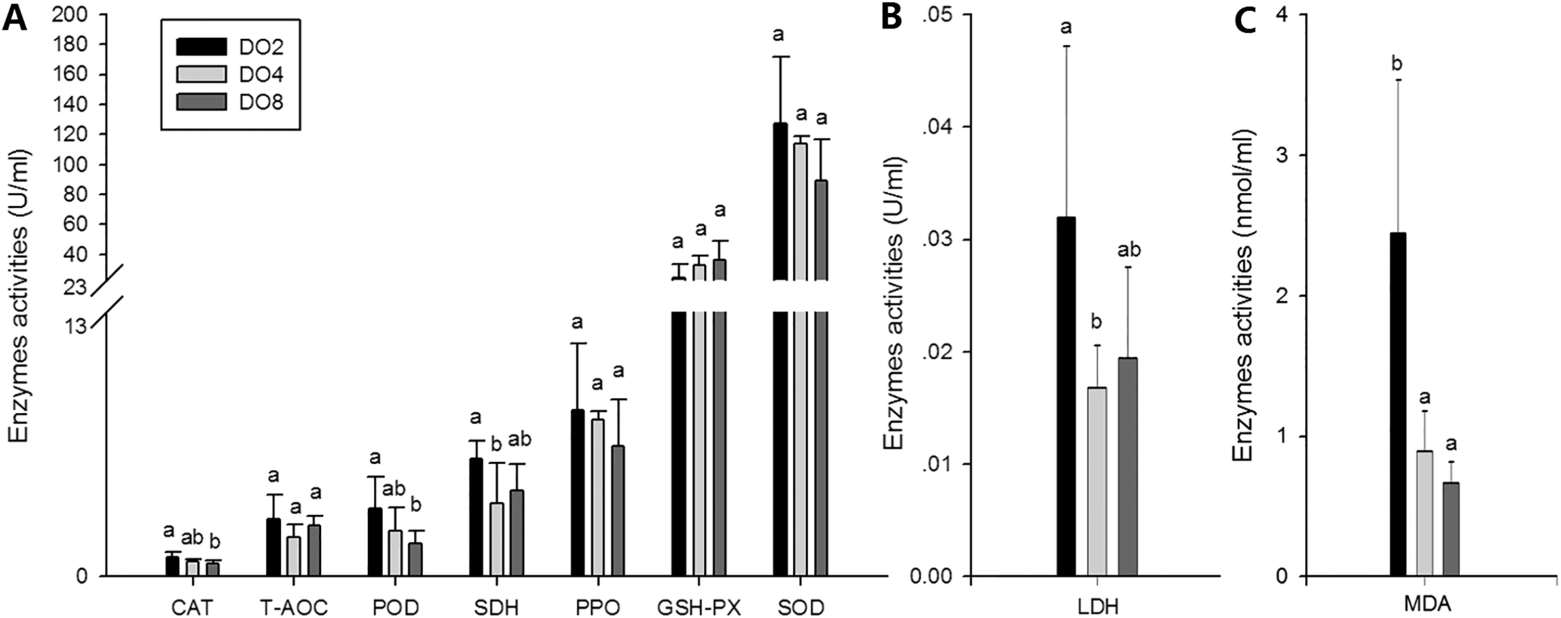
Activities of antioxidative enzymes in sea cucumber under different DO levels. (A) Activities of CAT, T-AOC, POD, SDH, PPO, GSH-PX, SOD in sea cucumber under different DO levels; (B) activity of LDH in sea cucumber under different DO levels; (C) content of MDA in sea cucumber under different DO levels.

To evaluate the oxidative responses of sea cucumber under hypoxia stress, nine indexes associated with oxidative responses were measured in this study (Fig. 6), including superoxide dismutase (SOD), glutathione peroxidase (GSH-PX), catalase (CAT), succinate dehydrogenase (SDH), lactate dehydrogenase (LDH), total antioxidant capacity (T-AOC), malondialdehyde (MDA), peroxidase (POD) and phenoloxidase (PPO). Generally, the activities of antioxidative enzymes were prompted under hypoxia. The activities of CAT, SDH, LDH and POD as well as the content of MDA significantly increased in severe hypoxia (2 mg/L). Activities of T-AOC, PPO GSH-PX, and SOD were not significantly changed under different environmental conditions. We speculate that hypoxic stress may cause an oxidative response in sea cucumbers. These changes in antioxidant enzyme activities in response to a hypoxic environment may be crucial for avoiding oxidative damage.

### Expressions of key responsive genes

**Figure 7:**
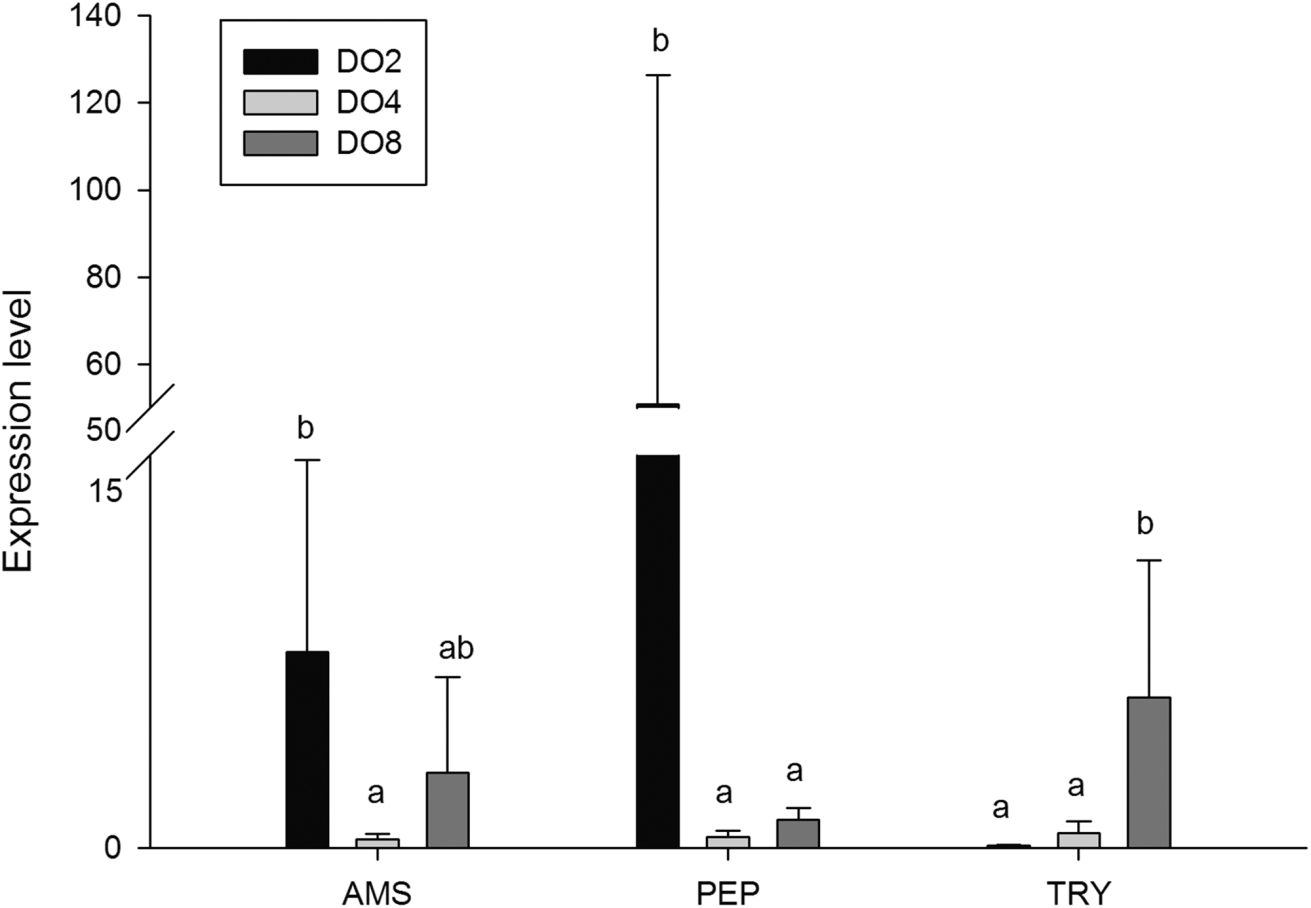
Real-time PCR analysis of the key genes related to digestive function.

**Figure 8:**
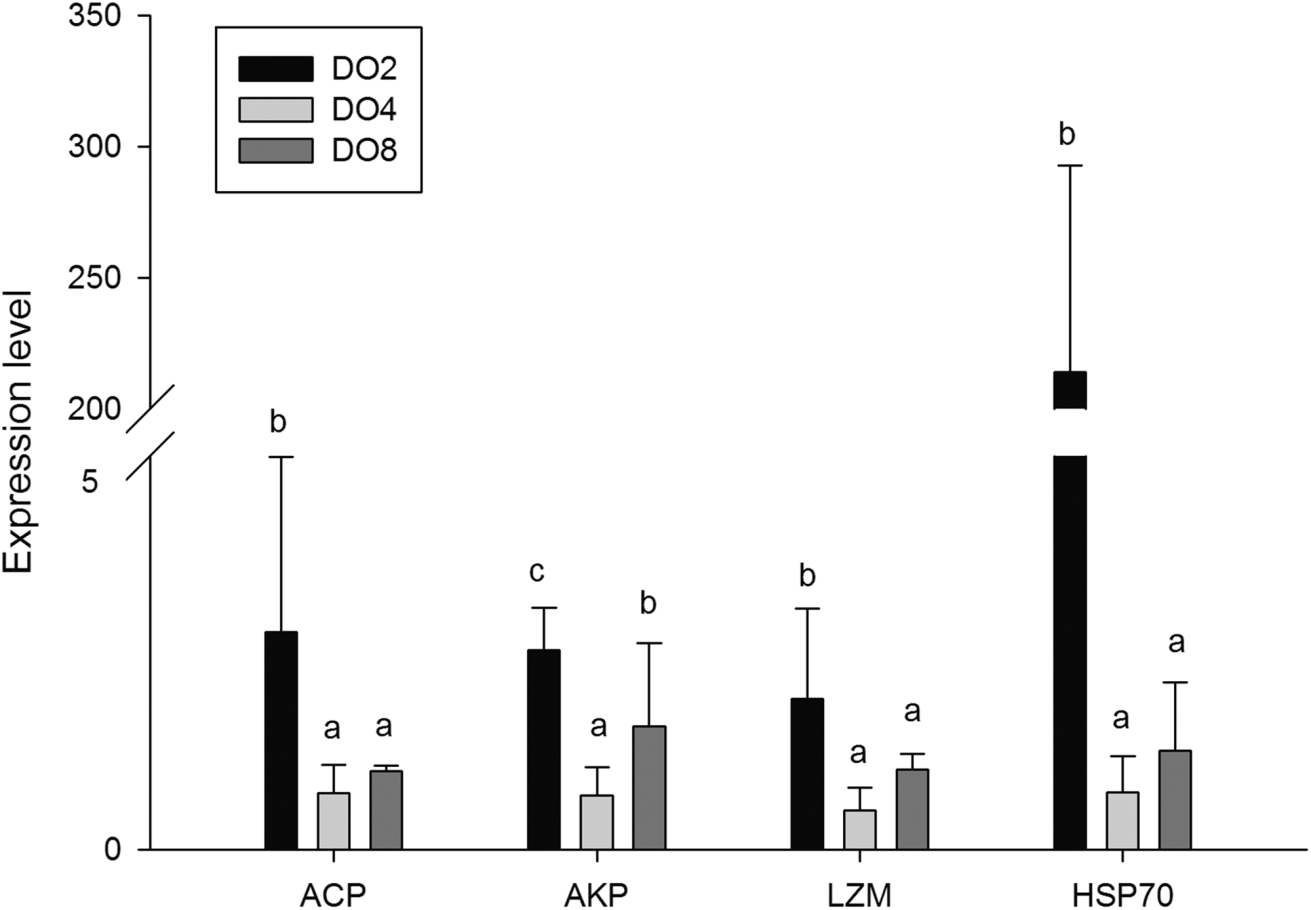
Real-time PCR analysis of the key genes related to immune defense.

**Figure 9:**
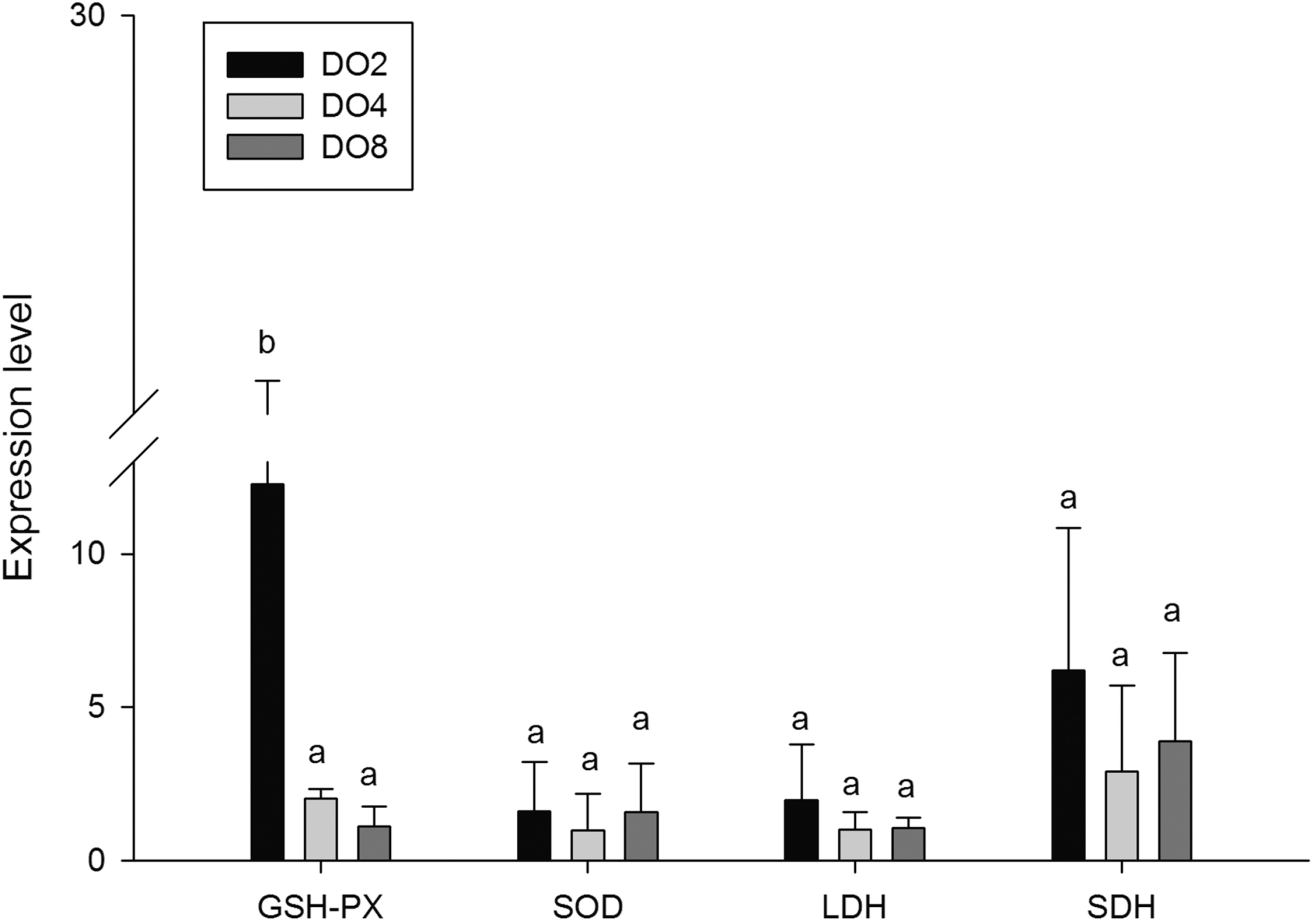
Real-time PCR analysis of the key genes related to oxidative response.

In order to investigate and verify the molecular mechanisms that trigger and regulate changes of digestion, oxidation, and immunity in sea cucumbers, 11 related genes were selected for further detection of the relative mRNA expression level using real-time PCR. According to the results, the *AMS* and *PEP* genes were expressed at a significantly high level under low DO exposure (2 mg/L), and the *PEP* gene was upregulated about 50-fold (Fig. 7). In addition, the *TRY* gene was significantly down-regulated about 70-fold under low DO exposure, indicating that hypoxic stress may have an influence on the digestive function of sea cucumber. Moreover, genes associated with immune defense were analyzed (Fig. 8). The four genes selected (*ACP, AKP, LZM,* and *HSP70*) all showed significantly high expression levels in hypoxia. Particularly, *HSP70* was up-regulated over 200-fold under severe hypoxia (DO 2 mg/L) compared with normoxic conditions. Thus, we speculated that immune defense was induced in sea cucumber after low DO exposure and *HSP70* could be a sensitive marker for hypoxia response. In addition, four genes (*GSH-PX, SOD, LDH*, and *SDH*) related with the oxidative response were also validated (Fig. 9). Among them, *GSH-PX* was significantly upregulated about 10-fold under severe hypoxia. Though the activities of LDH and SDH were significantly increased under hypoxia, *LDH* and *SDH gene* expression levels were not significant, as well as the *SOD* gene. The real-time PCR data corresponds to enzyme activity, indicating that complex molecular and physiological mechanisms are involved in the response to hypoxic stress.

## Discussion

### Respiratory related changes under hypoxia

Respiration is often quantified by measuring OCR (Yang et al., 2006). The significant effect of different DO levels and body weight on oxygen consumption was demonstrated in the present study. Results showed that OCRs decreased with the decline of the DO level, and OCR of the high weight group decreased more rapidly than the low weight group. In previous studies, decreased oxygen consumption was observed in several aquatic animals under environments of low DO levels, including holothurian *Stichoplls parvimensis* (Dimock, 1977), bivalve *Arctica islandica* (Taylor & Brand, 1975), *Corbicula fluminea* (McMahon, 1979), fish *Cichlasoma urophthalmus* (Palacios & Ross, 1986), *Acipenser transmontanus* (Crocker & Cech, 1997), and *Scophthalmus maximus* (Pichavant et al., 2001). Moreover, the increased OCRs were observed in *Pomatoschistus minutus* (Petersen & Petersen, 1990), *C. urophthalmus* (Palacios & Ross, 1986) and *A. islandica* (Taylor & Brand, 1975) with higher weights. Our results are consistent with previous studies mentioned above and we speculated that the decrease of OCR is an adaptive response to low DO, which would depress energy consumption and reduce the accumulation of reactive oxygen species (ROS) and related deleterious effects.

Moreover, previous research illustrated that the respiratory tree is the primary respiratory organ of the sea cucumber and cutaneous respiration is the other major form of respiration (Choe, 1963). In this study, white and ulcerated spines were observed in some sea cucumbers during the early stage of hypoxia stress, and some individuals eviscerated internal organs. Due to the loss of the respiratory tree, the body wall will perform respiration for the whole animal, which might have an effect on the respiratory progress as well as immune functions (Choe, 1963). Furthermore, hypoxia stress caused diffused internal organs and changed enzyme activities and metabolism process in sea cucumber. These changes would also have an influence on the respiratory process, especially for the sea cucumbers in the natural environment. Thus, we speculated that in a hypoxic environment, the respiration of sea cucumbers would be suppressed because of evisceration, the damaged body wall, morphological changes of respiratory tree and other undescribed physiological alternations. The suppression would be much more serious for the sea cucumbers live in natural environment.

### Digestive changes under hypoxia stress

The activity of digestive enzymes is one of the most commonly used indicators of ingestive capability, nutritional biochemistry, and physiological status (Fu et al., 2005). It was reported that the activities of digestive enzymes in *A. japonicus* were affected by various factors, including temperature, salinity, intestinal microflora, food intake, and gut development (Gao et al., 2009). Thus, it is of great significance to understand the physicochemical property and activity of enzymes. A previous study showed that total amylase and TRY activity were significantly higher under low salinity in white shrimp *Litopenaeus vannamei* (Li et al., 2008). AMS activity was significantly affected in sea cucumber by water temperature and duration (Gao et al., 2009). AMS, LPS and TRY activities in white shrimp *L. vannamei* decreased continuously under conditions of cyclic serious/medium hypoxia versus normoxia, especially the most sensitive trypsin (Han et al., 2017). In the present study, decreased activities of LPS and TRY were observed with the decline of DO levels, and *TRY* gene is significantly down-regulated about 70-fold in sea cucumbers when DO of ambient environment is insufficient. Moreover, the activity of AMS and *AMS* genes expressed at a significantly high level under low DO exposure (2 mg/L). Furthermore, the *PEP* gene significantly upregulated about 50-fold under severe hypoxia compared with normoxic condition. Thus, we speculated that sea cucumbers would choose nutrient materials which are easy to digest, such as amylum and protein, rather than lipid, to quickly gain energy for adaptation to a hypoxic environment. It is noteworthy that PEP and TRY are both proteases and could degrade the protein into polypeptides, but the tendency of these two proteases is not the same. As described previously, digestive enzymes are secreted from enterocytes of the foregut and midgut in *A. japonicus* (Cui, Dong & Lu, 2000). Moreover, the optimal conditions for PEP are acidic, while alkaline conditions are more suitable for TRY (Li et al., 1995). Therefore, we speculated that the reason of opposite tendency in these two proteases is related with different digestive states and acid–base status in sea cucumbers. In general, the level of DO influences the activity of digestive enzymes, indicating that hypoxia may have an effect on digestive function and may change the nutritional requirements and acid–base status in sea cucumbers. Further study is needed to verify the hypothesis.

### Immune defense under hypoxic stress

It is commonly believed that the immune response of *A. japonicus* is a typical non-specific immune response (innate response) (Wang et al., 2015). After phagocytic processes, the first line of the body’s internal defense, ACP, AKP and LZM could assist in the complete degradation of exogenous substances (Canicattí, 1990; Janeway & Medzhitov, 2002). Moreover, phosphatase plays an important role in dephosphorylation reactions, particularly in performing signal transduction, physiological metabolism, and environmental adaptation (Kong et al., 2012). Thus, in the present study, the enzyme activities and gene expressions of ACP, AKP, and LZM, and a well-known immune-related gene, *HSP70* were measured in sea cucumbers under different DO levels. Results showed that the expression of *ACP* and *AKP* genes were increased under severe hypoxia and the activity of ACP shared the same tendency. The mutual increases in both ACP and AKP suggest defense against foreign materials is enhanced and metabolic intensity is improved to provide more energy (Zang et al., 2012). We speculated that sea cucumber could adapt to the hypoxic pressure in the external environment by consuming more ATP in metabolism processes, and the inorganic phosphoric acid required for ATP synthesis can be produced through hydrolysis of the phosphate ester by ACP and AKP (Zheng et al., 2014). Moreover, hypoxia stress may lead to a high level of exogenous harmful substances and induce immune defense in sea cucumber, resulting in an increase in ACP and AKP activity.

Lysozyme is an important innate immunity factor that kill bacteria, thereby preventing bacterial infection, and commonly exists in the coelomocyte and coelomic fluid of echinoderms (Canicatti & Roch, 1989; Shimizu et al., 1999; Kong et al., 2012). Previous study showed that when the environmental factors changed beyond the optimum conditions for organisms, LZM levels would correspondingly change. For example, increased LZM levels were observed in Atlantic halibut under the elevated culture temperature (Langston et al., 2002). Moreover, changes of salinity would induce the activity of LZM in coelomic fluid of sea cucumber (Zheng et al., 2014). In the present study, the *LZM* gene was expressed at a significantly high level in the respiratory tree of sea cucumber under stress from hypoxia, indicating that LZM is affected by hypoxia and could lead to a high immunocompetence in sea cucumbers. The various microbiome influenced by different DO levels may result in a diverse acceleration of enzyme activities.

Heat shock proteins were reported to serve as another important defense in protecting organisms through repairing damaged proteins denatured by stressors and have significant effects on stress resistance and life span (Sorensen & Loeschcke, 2001; Tovar-Ramírez et al., 2010). According to research published previously, the structures and activities of some proteins may be changed under stress, and HSPs would be upregulated to refold denatured proteins (Feder & Hofmann, 1999; Morimoto & Santoro, 1998). Thus, further aggregation and precipitation was prevented. In previous studies, *HSP70* was demonstrated to be induced by hypoxia stress in Nile tilapia *Oreochromis niloticus*, Korean rockfish *Sebastes schlegeli* and oriental river prawn *Macrobrachium nipponense* (Delaney & Klesius, 2004; Mu et al., 2013; Sun et al., 2016). Moreover, *HSP70* was used as a bioindicator of thermal stress in the sea cucumber, and the expression pattern depended on the varied temperature treatments (Dong, Ji & Dong, 2007). In this study, *HSP70* was upregulated over 200-fold in sea cucumber after hypoxia stress, indicating that *HSP70* responses are sensitive to low DO exposure and could be a biomarker to characterize severe stresses. In conclusion, increased *ACP*, *AKP*, and *LZM* revealed an enhanced ability to synthesize more enzymes and may be used to strengthen the ability to meet the requirements of metabolism in hypoxic stress and to attenuate oxidative damage. Therefore, we speculated that an immune defense was induced in sea cucumber after low DO exposure.

### Oxidative responses under hypoxia stress

Previous studies have demonstrated that hypoxia affects the oxidative status of aquatic animals, such as shrimp *L. vannamei* (Liu et al., 2015; Parrilla-Taylor & Zenteno-Savin, 2011), crab *Paralomis granulosa* (Romero, Anasldo & Lovrich, 2007; Romero et al., 2011), scallop *Chlamys farreri* (Chen et al., 2007) and fish *Sparus aurata* (Pérez-Jiménez et al., 2012). To survive under hypoxia, an adaptive strategy is used to reduce the level of ROS. As highly reactive molecules, ROS can oxidize cellular components, and this leads to oxidative stress when an imbalance occurs between producing and removing ROS (Halliwell & Gutteridge, 2001; Kong et al., 2012). Normally, organisms are protected against the deleterious effects of ROS, which include superoxide (O_2_^−^), hydrogen peroxide (H_2_O_2_), hydroxyl free radical (OH^−^), and singlet oxygen (^1^O_2_), by a complex antioxidant system constituted by enzymatic and non-enzymatic detoxification mechanisms (Elstner, 1982; Smirnoff, 1993; Van Breusegem, Van Montagu & Inzé, 1998). The mechanism for sea cucumber to counteract oxidative damage includes enzymatic and non-enzymatic antioxidant defenses. Antioxidant enzymes are the first line of defense against ROS, including SOD, CAT, GSH-PX. Thus, the activities of enzymes, such as SOD, CAT, and MDA, could be changed when the organism is faced with stress.

Changes in CAT and SOD activity eliminate ROS and detoxified O_2_^−^ and H_2_O_2_, respectively (Hermes-Lima, Storey & Storey, 1998). While their activities are related to the status of the organisms affected by environmental factors (Winston & Di-Giulio, 1991). In the present study, the level of CAT activity was increased under severe hypoxia, indicating that more radicals were involved in the reactions and hypoxia stress resulted in radicals accumulating to a higher level in sea cucumbers. Although the change of SOD was not significant in our study, it was reported to be suppressed in muscle of sea cucumber under hypoxia (Li et al., 2016). Therefore, the adaptive strategies of sea cucumber in hypoxic environment could be partially explained by the changing activities of antioxidative enzymes, such as CAT and SOD, for scavenging the radicals produced to a certain extent, thus protecting from aging and cellular damage.

Oxidative enzymes like SDH and LDH could reflect cellular metabolism as well as the utilization of intermediates in the Krebs cycle and related metabolic pathways (Stokes, Vitale & Morgan, 1979). SDH activity is highly correlated with Krebs cycle flux and mitochondrial respiratory capacity, thus it is considered as a standard marker of relative oxidative capacity (Bass et al., 1969; Newsholme & Leech, 1984). Moreover, the activity of LDH is measured as an enzymatic marker of glycolytic capacity. The marked increases in SDH and LDH activities observed in our study suggests an alternative shift in energy metabolism from aerobic metabolism to anaerobic glycolysis may due to hypoxia-induced mitochondrial damage.

MDA, which is a measure of the terminal products of lipid hydroperoxides, could reflect stress by oxyradicals in organisms and is a principal and well-studied biomarker of oxidative stress damage (Del Rio, Stewart & Pellegrini, 2005; Zang et al., 2012). Previous studies have shown that MDA accumulation is positively related to the level of oxidative stress (Shi et al., 2005; Sun et al., 2008). Moreover, significantly increased content of MDA was observed in *Carassius auratus* under hypoxia (Zhao et al., 2017). In the present study, the content of MDA in coelomic fluid from sea cucumber increased about twofold under hypoxia, suggesting that hypoxia stress caused lipid peroxidation resulting from oxidative stress. POD and PPO, which catalyze the oxidation of phenols to quinones, were measured in the present study (Vaughn & Duke, 1984; Thipyapong, Hunt & Steffens, 1995). POD, which could play an essential role during phagocytosis and for destroying invading microorganisms in invertebrates, shows a marked elevation of activity in hypoxia (Xing, Zhan & Zhou, 2002). However, the increase of PPO activity was not significant when coping with stress from hypoxia in this study. The results suggest evidence of the participation of these enzymes during the hypoxia stress process.

Glutathione peroxidase (GSH-PX) was measured as a marker of cellular antioxidant enzyme activity because of its capacity to detoxify H_2_O_2_ and organic hydroperoxides (Halliwell & Gutteridge, 2001). A previous study stated that GSH-PX activity of hemolymph supernatant in crabs was increased after hypoxia exposure (Qin et al., 2016). Moreover, significantly increased GSH-PX activity in the heart and lungs, and significantly decreased activity in the liver were observed in rats under hypoxia (Nakanishi et al., 1995). In the present study, no significant difference of GSH-PX activities was found in the three groups but the *GSH-PX* gene was significantly upregulated about 10-fold in hypoxia. The reason might be explained partially by a different oxidative response in different tissues under hypoxia stress. The T-AOC, which is measured to explain antioxidant defenses, showed elevated activity in hypoxia but not significantly. These two indexes might not be very sensitive to hypoxia. We speculated that oxidative responses were mainly made by other enzymes in sea cucumber. The changes of oxidative enzymes mentioned above indicated that the response of the antioxidant system in *A. japonicus* was prompt under hypoxia stress. These changes could help to avoid oxidative stress and are supposed to be an adaptive mechanism that allows sea cucumber to tolerate exposure to a hypoxic environment.

## Conclusion

In the present study, we demonstrated how enzyme activities, key genes, OCR, and tissue morphology change in sea cucumber under hypoxia. After hypoxia exposure, the activities of enzymes and expression of key genes related to digestion function, immune defense, and oxidative response were changed significantly. With the decline of DO levels, oxygen consumption rate was significantly decreased in the high weight group and the downtrend was more rapid than in the low weight group. Moreover, the internal organs were negatively impacted. All the results showed that respiration was suppressed, digestive function changed, and antioxidant system and immune defense of sea cucumber was prompted under hypoxia. In conclusion, responses of sea cucumbers to hypoxia caused a cascade of molecular, morphological and physiological processes, and we suppose that *A. japonicus* developed strategies to endure the diminution of oxygen availability. Evidence on the mechanism that *A. japonicus* uses to cope with hypoxic stress is provided by our results for future study.

## Supplemental Information

10.7717/peerj.4651/supp-1 Supplemental Information 1 Raw data for the thickness of the respiratory tree.

10.7717/peerj.4651/supp-2 Supplemental Information 2 Raw data for the activities of key enzymes.

10.7717/peerj.4651/supp-3 Supplemental Information 3 Raw data for expression levels of the key genes.

10.7717/peerj.4651/supp-4 Supplemental Information 4 Raw data for oxygen consumption rate of sea cucumber under hypoxia stress.

10.7717/peerj.4651/supp-5 Supplemental Information 5 Different behavior of sea cucumbers under hypoxia stress. A. Sea cucumbers with stretched tentacles; B. Distorted sea cumbers with ulcerated skin; C. Shrunken sea cucumber; D. Edematous sea cucumber; E. Sea cucumber at mid-eviscerated stage. The photos were taken by Da Huo.

10.7717/peerj.4651/supp-6 Supplemental Information 6 Different morphology of sea cucumbers under hypoxia stress. A. Discolored sea cumbers; B. Whitening spines; C. Diffused respiratory trees and intestines; D. Healthy sea cucumber with straight spines. The photos were taken by Da Huo.

10.7717/peerj.4651/supp-7 Supplemental Information 7 Changes of internal organs in sea cucumber under short-term hypoxia. A. internal organs under normal conditions; B. internal organs under hypoxia. The photos were taken by Da Huo.

10.7717/peerj.4651/supp-8 Supplemental Information 8 Selected genes and their primer sequences used for real-time PCR.

## Additional Information and Declarations

### Data Availability

The following information was supplied regarding data availability: The raw data are provided as Supplemental Files.
