# Supplementary figures and images for: Impact of hypoxia stress on the physiological responses of sea cucumber Apostichopus japonicus: respiration, digestion, immunity and oxidative damage
Source: PeerJ. 2018 Apr 27;6:e4651. doi: 10.7717/peerj.4651 (PMC5926553; doi:10.7717/peerj.4651)

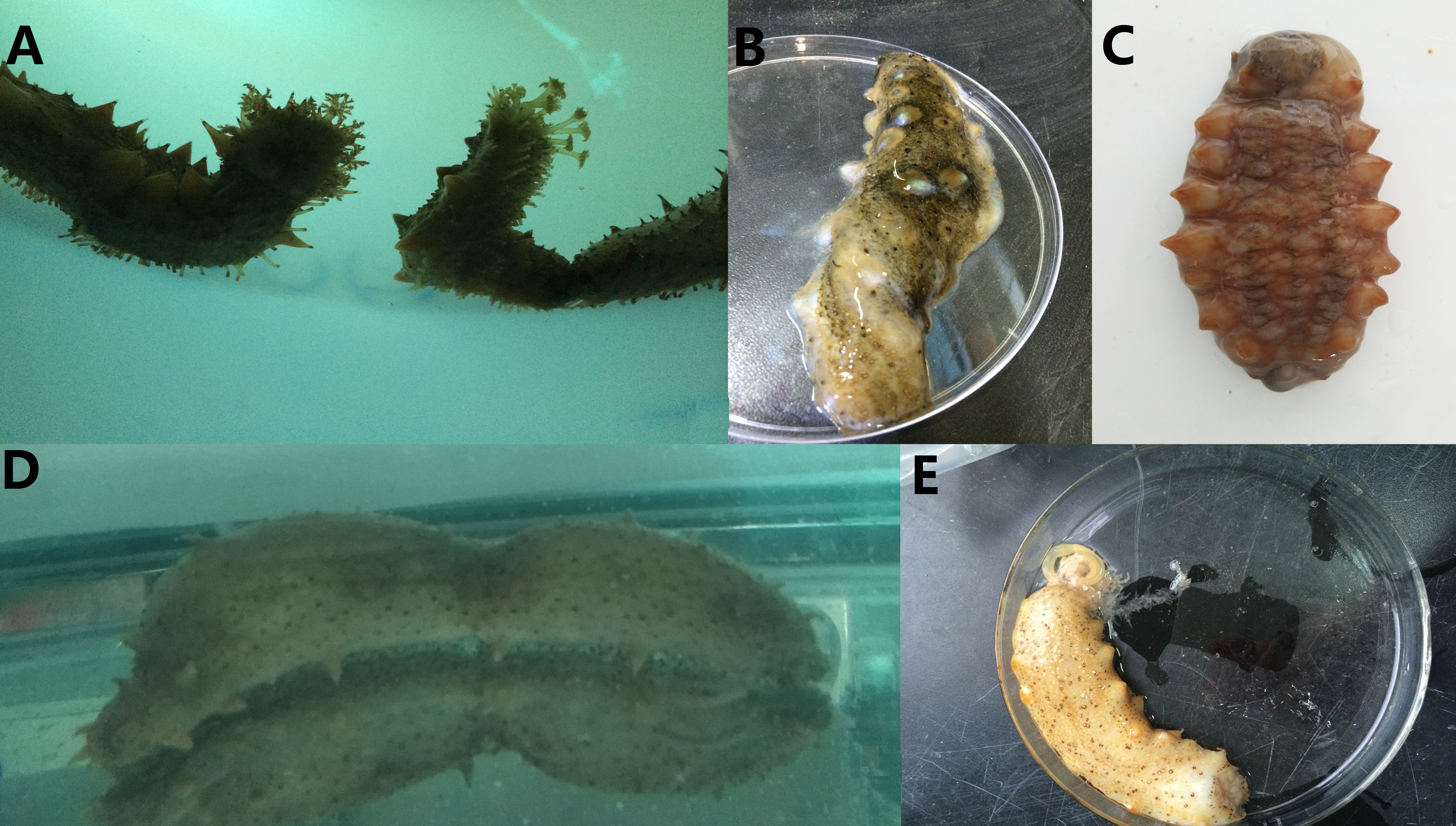

Supplement: Supplemental Information 5 — A. Sea cucumbers with stretched tentacles; B. Distorted sea cumbers with ulcerated skin; C. Shrunken sea cucumber; D. Edematous sea cucumber; E. Sea cucumber at mid-eviscerated stage. The photos were taken by Da Huo. [file peerj-06-4651-s005.png]

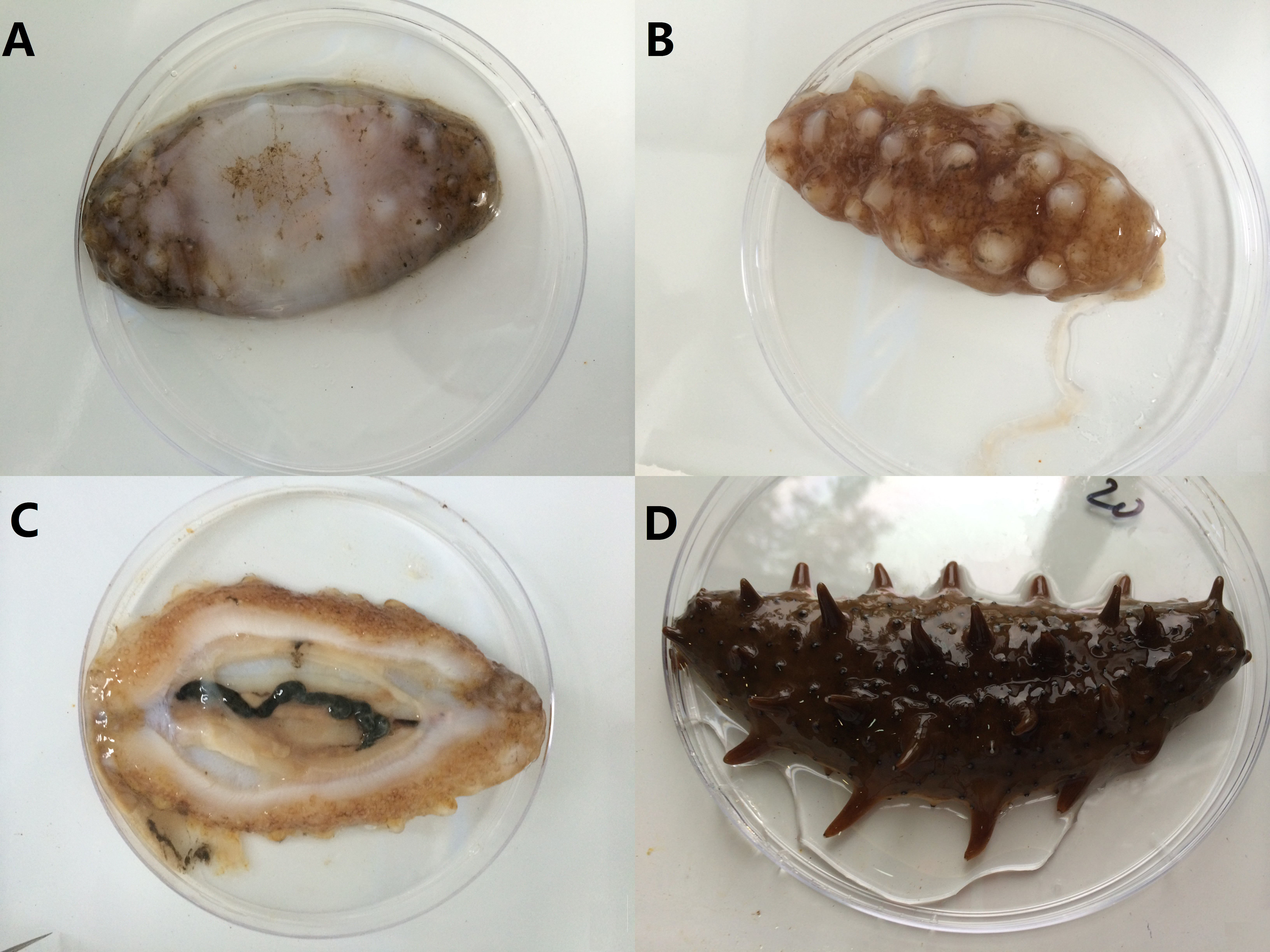

Supplement: Supplemental Information 6 — A. Discolored sea cumbers; B. Whitening spines; C. Diffused respiratory trees and intestines; D. Healthy sea cucumber with straight spines. The photos were taken by Da Huo. [file peerj-06-4651-s006.png]

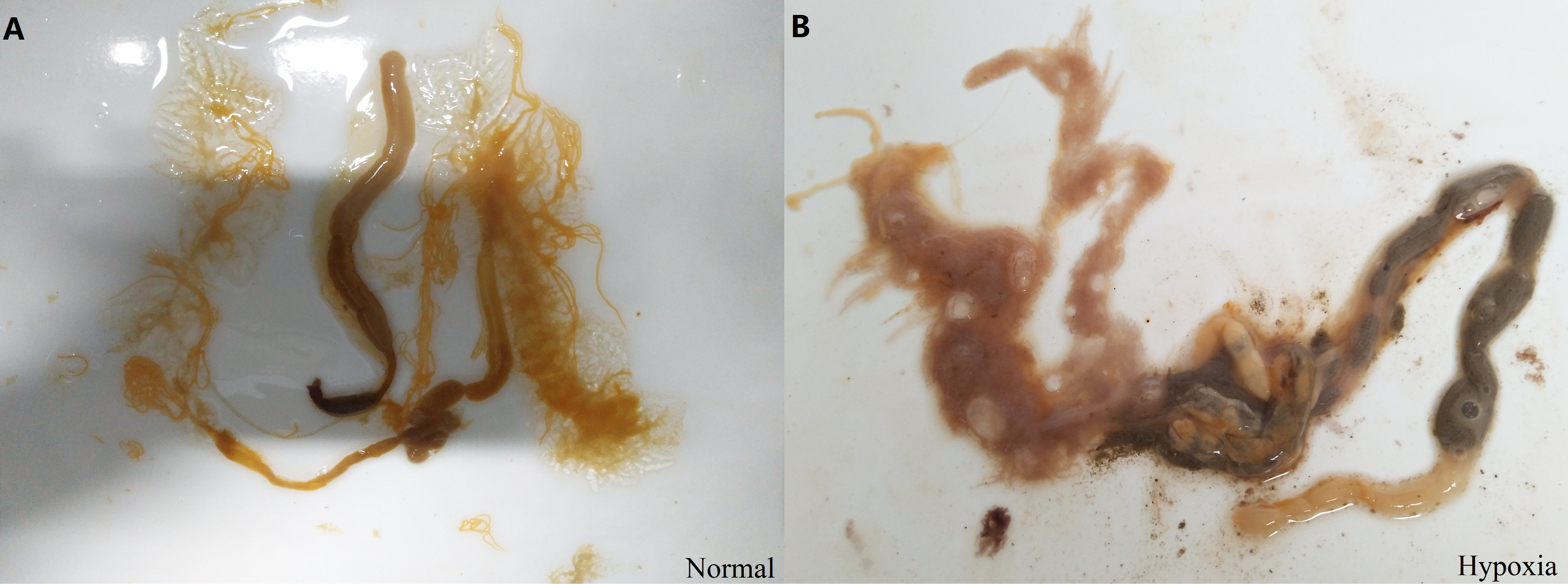

Supplement: Supplemental Information 7 — A. internal organs under normal conditions; B. internal organs under hypoxia. The photos were taken by Da Huo. [file peerj-06-4651-s007.png]
