# Supplementary material for: Impact of hypoxia stress on the physiological responses of sea cucumber Apostichopus japonicus: respiration, digestion, immunity and oxidative damage
Source: PeerJ. 2018 Apr 27;6:e4651. doi: 10.7717/peerj.4651 (PMC5926553; doi:10.7717/peerj.4651)
Supplement: Supplemental Information 8 [file peerj-06-4651-s008.docx]

Table S1 Selected genes and their primer sequences used for real-time PCR

| Gene name | Abbreviation | Primer sequence (5′ to 3′) |
| --- | --- | --- |
| L-lactate dehydrogenase | LDH | F: AGCGGAACAAACTTGGACAC |
|  |  | R: CCAAAGTGGTACGCTGGAAT |
| succinate dehydrogenase | SDH | F: TGGTCTTGGATGCCCTAGTT |
|  |  | R: GCCAGAGTGTTGGTTCCATT |
| glutathione peroxidase | GSH-PX | F: ATAGGCGTACCATCGAACCA |
|  |  | R: CGCTGGATCACCAATTTCTT |
| superoxide dismutase | SOD | F: TCTGAAGGAGGGCTGTCAGT |
|  |  | R: AACTACGCCTTGGTGGTCAG |
| alkaline phosphatase | AKP | F: GCCTACCTTACCGGTGTCAA |
|  |  | R: TGTCCGCCCTTACACCTATC |
| lysozyme | LZM | F: AGGCTACTGGCAGGATGCTA |
|  |  | R: TTGCGTACCGTGCCATATAA |
| acid phosphatase | ACP | F: TGTCAATCAGTGGGCTCAGA |
|  |  | R: AGACCTGAGACGATGCTGGT |
| peptidase | PEP | F: CCAGAATAACGCTGGAAACC |
|  |  | R: GTTGCTGGGTTGTTTGAGGT |
| trypsin | TRY | F: GTCGCTTTACACGATGAGCA |
|  |  | R: TTCGATCCAGTCCGAGAAAC |
| alpha-amylase | AMS | F: ATATGGATTCGGTGGAGTGC |
|  |  | R: TTCGTCACCACCTCTGGAAT |
| heat shock protein 70 | HSP70 | F: GTCTCTGGGTCTTGAAACAGC |
|  |  | R: CTATCTTAGGAACGCCTCGTG |
| β-actin | actin | F: GGGCCAGCATTCCACTAATA |
|  |  | R: TGCCACGGGAGGTACTAAAC |
